# Combustion of dried animal dung as biofuel results in the generation of highly redox active fine particulates

**DOI:** 10.1186/1743-8977-2-6

**Published:** 2005-10-04

**Authors:** Ian S Mudway, Sean T Duggan, Chandra Venkataraman, Gazala Habib, Frank J Kelly, Jonathan Grigg

**Affiliations:** 1 Lung Biology: Pharmaceutical Science Research Division, School of Biomedical & Health Sciences, King's College London, Franklin-Wilkins Building, 150 Stamford Street, London, SE1 9NH, UK; 2 Department of Chemical Engineering, Indian Institute of Technology, Bombay, Powai, Mumbai-400 076, India; 3 Division of Child Health, Department of Immunology, Infection and Immunity, University of Leicester, PO Box 65, Leicester

## Abstract

**Background:**

The burning of biomass in the developing world for heating and cooking results in high indoor particle concentrations. Long-term exposure to airborne particulate matter (PM) has been associated with increased rates of acute respiratory infections, chronic obstructive lung disease and cancer. In this study we determined the oxidative activity of combustion particles derived from the biomass fuel dung cake by examining their capacity to deplete antioxidants from a model human respiratory tract lining fluid (RTLF). For comparison, the observed oxidative activity was compared with that of particles derived from industrial and vehicular sources.

**Results:**

Incubation of the dung cake particle suspensions in the RTLF for 4 h resulted in a mean loss of ascorbate of 72.1 ± 0.7 and 89.7 ± 2.5% at 50 and 100 μg/ml, respectively. Reduced glutathione was depleted by 49.6 ± 4.3 and 63.5 ± 22.4% under the same conditions. The capacity of these samples to deplete ascorbate was in excess of that observed with diesel or gasoline particles, but comparable to that seen with residual oil fly ash and considerably in excess of all three control particles in terms of glutathione depletion. Co-incubation with the metal chelator diethylenetriaminepentaacetate inhibited these losses, whilst minimal inhibition was seen with superoxide dismutase and catalase treatment. The majority of the activity observed appeared to be contained within aqueous particle extracts.

**Conclusion:**

These data demonstrate that biomass derived particles have considerable oxidative activity, largely attributable to their transition metal content.

## Background

Approximately two billion people in the developing world use biomass fuels such as wood, crop-waste and dried animal dung, as their major source of domestic energy [1,2]. The burning of this material in open fires and stoves results in high concentrations of particulate matter (PM), carbon monoxide, nitrogen dioxide, as well as volatile and semi-volatile organic species in the indoor environment [3,4]. In homes where biomass burning occurs airborne particle concentrations are far in excess of those found in homes where it is not used as a heating/cooking source, with 24 h average PM_10 _concentrations ranging between 200 and 5000 μg/m^3 ^depending on the fuel type, stove and ventilation [5,6]. These concentrations are far in excess of the level considered safe for PM_10 _in outdoor air – 150 μg/m^3 ^24 h average [7].

An increasing body of evidence has linked exposure to indoor pollutants with increased rates of respiratory morbidity and mortality. Women and children exposed to high indoor PM concentrations have significantly increased rates of acute respiratory infections (ARI) [5,8-10], and women cooking over biomass fires for extensive periods have an enhanced risk of chronic obstructive pulmonary disease (COPD) and lung cancer [11,12]. In a recent study we found that lower airway cells from women and children exposed to biomass smoke contained significantly more carbonaceous material than age-matched subjects exposed to fossil fuel derived PM_10 _[13]. Whilst enhanced particle deposition in the airways is clearly important, the chemical and physical characteristics of airborne particles that contribute to their toxicity have not been firmly established, though particle size and surface area [14], as well as acidity and composition [15,16] have all been mooted as important determinants in this regard.

The capacity of inhaled PM to elicit damaging oxidation reactions in the lung and systemic circulation may account for many of the toxic responses observed in exposed individuals [17]. Certain transition metal components of PM are capable of catalysing oxidation reactions [18], as are quinones [19] resulting in the production of reactive oxygen species. An excessive production of these species can activate redox sensitive transcription factors regulating the expression of pro-inflammatory cytokines, leading to inflammation and tissue injury [20]. Measuring the capacity of PM to oxidise physiologically relevant molecules can therefore be used to provide a biologically relevant index of activity, integrating PM size, surface area and composition.

In this study we evaluated the oxidative activity of PM samples derived from the controlled burning of cow-dung cake using a traditional Indian cooking stove. These assessments were based on the capacity of dung cake particulate suspensions to deplete physiologically relevant antioxidants, ascorbate (AA), urate (UA) and reduced glutathione (GSH) from a synthetic model of human respiratory tract lining fluid. Previously we have used this model to investigate the nature of gaseous and particulate pollutant-antioxidant interactions at the air lung interface [21,22]. In addition we examined the extent to which any observed activity could be attributed to the metal or organic content of the dung cake particles.

## Results

**Figure 1:**
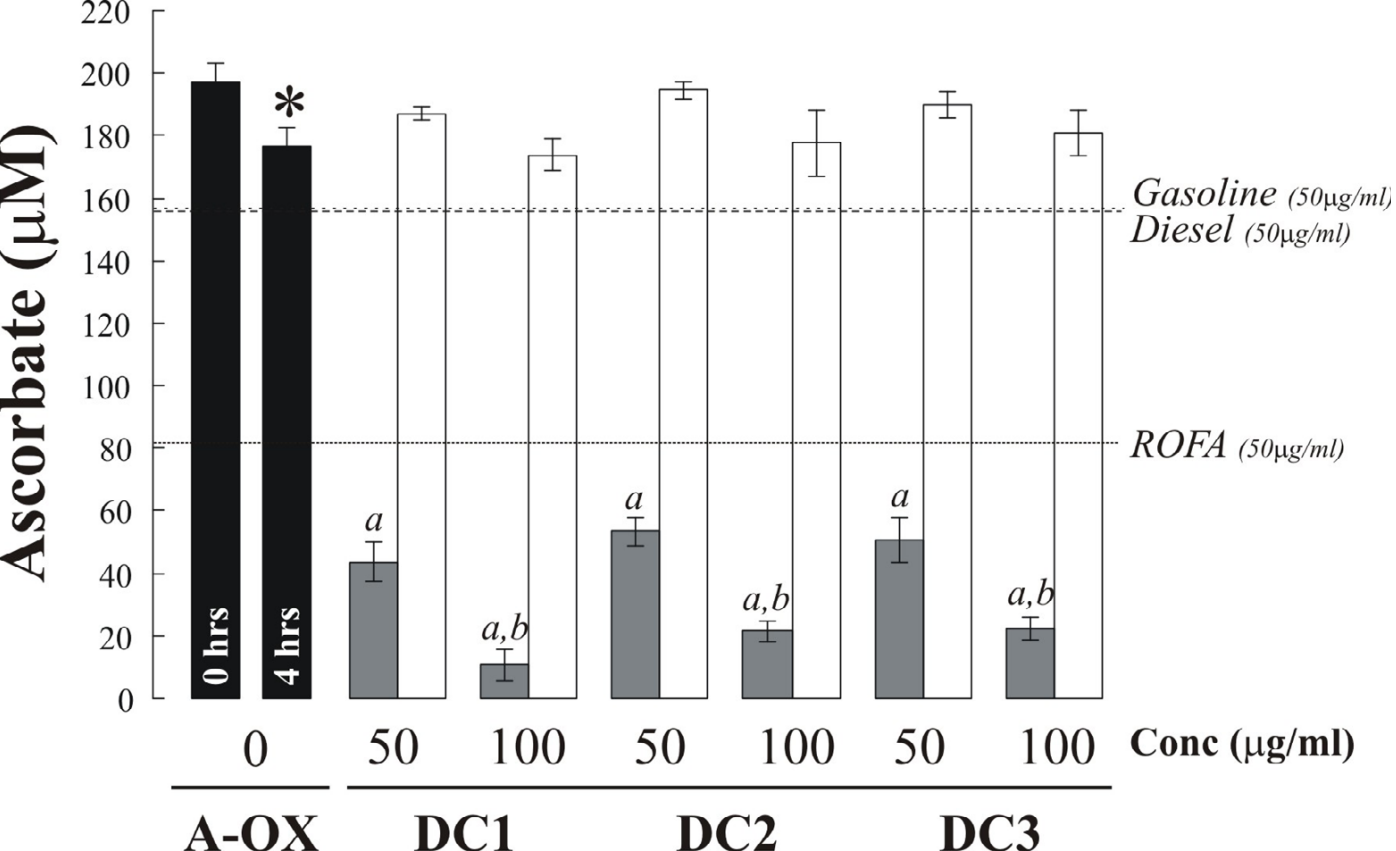
*AA remaining in synthetic RTLF following a 4 h incubation with three separate dung cake (DC) samples at 50 and 100 μg/ml (grey bars)*The impact of co-incubation with the transition metal chelator DTPA (200 μM) is illustrated by the white bars. Pre (0 hr) and post (4 hr) AA concentrations in the particle-free controls are illustrated in the black filled bars. The losses observed with 50 μg/ml doses of fresh diesel and gasoline PM_0.1–2.5_, as well as ROFA are also illustrated. Data represent the mean (SD) of 3 separate experiments: '*a*' – indicates that AA concentrations after the 4 h incubation were significantly lower (P < 0.05) than the 4 h particle free control values; *'b*' illustrates that the losses observed at 100 μg/ml are significantly greater than those seen at the lower 50 μg/ml concentration. The '*' illustrates a significant loss of AA in the particle free control over the 4 h incubation period.

Three Teflon filters were supplied containing 0.86 (DC1), 1.20 (DC2) and 1.14 mg (DC3) of PM_2.5_. Particle suspensions prepared from each of the three filters depleted AA from the synthetic RTLF in a dose dependent manner with a mean loss of 72.1 ± 0.72 and 89.7 ± 3.7% AA at 50 and 100 μg/m^3 ^respectively relative to the 4 h particle free control concentration. This loss of AA was significantly greater than that associated with an equal mass (50 μg/m^3^) of diesel (6.4%), gasoline (5.2%) or residual oil fly ash (ROFA) (53.9%) particles (Figure 1). All of the tested PM samples were PM_2.5_, apart from the diesel and gasoline samples that were collected using a high volume cascade impactor as PM_0.1–2.5_. Of these particles ROFA had the smallest median diameter, 0.07–0.08 μm [23], with diesel and gasoline PM samples in the range 0.1–0.2 μm (Thomas Sandström, personal communication), with the dung cake samples displaying the largest median aerodynamic diameter, 0.6–0.8 μm [4]. These data further emphasise the reactivity of the DC samples, taking into account their lower surface area per unit mass, compared with the control PM samples. These AA losses were completely inhibited when the dung cake particles were co-incubated with 100 μM DTPA (Figure 1).

**Figure 2:**
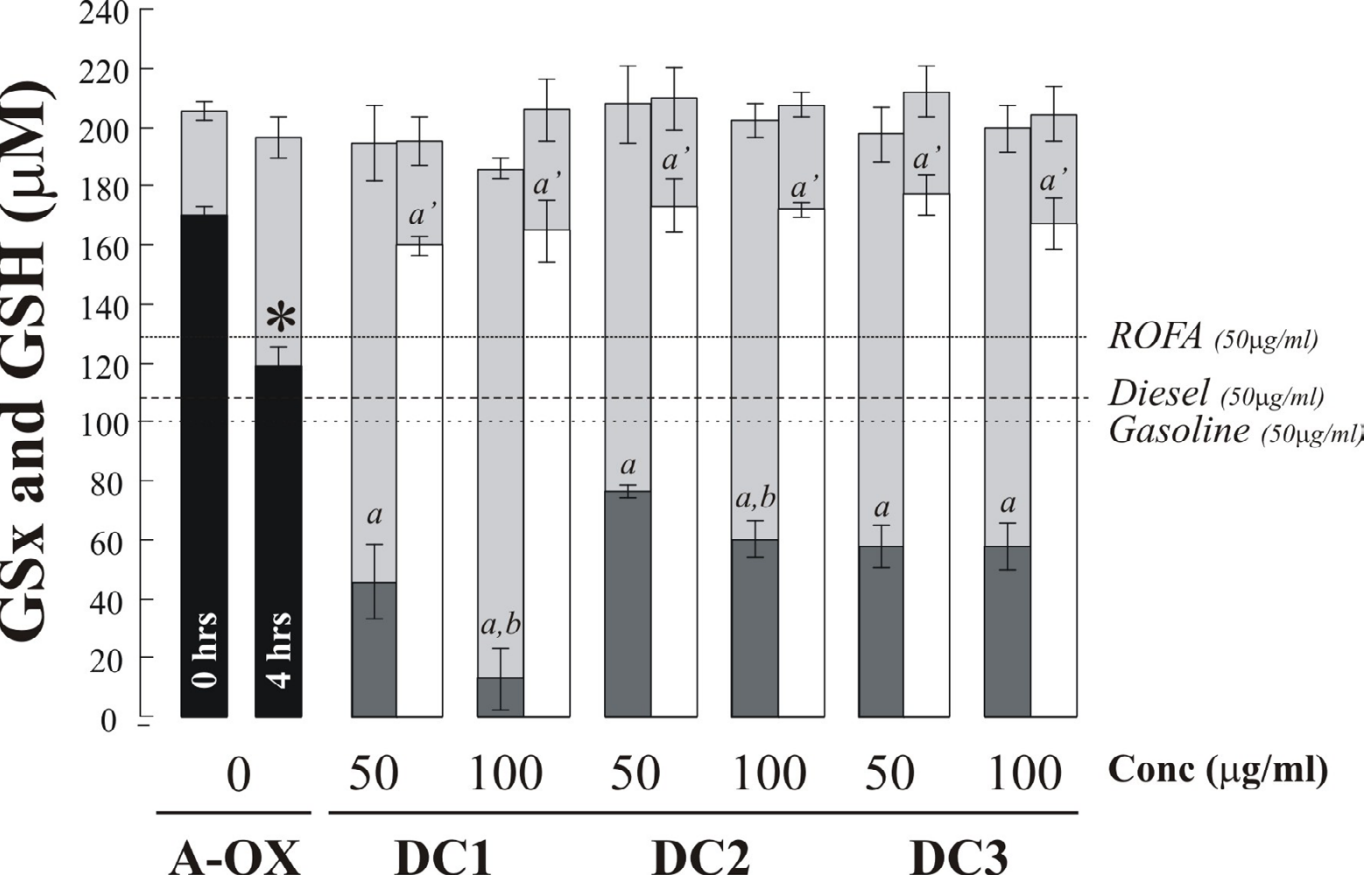
*Total (GSx) and reduced glutathione (GSH) remaining in synthetic RTLF following a 4 h incubation with three separate dung cake (DC) samples at 50 and 100 μg/ml*. Reduced glutathione concentrations after 4 h are illustrated by dark grey bars, whilst the corresponding total glutathione concentration (GSx) are shown in light grey beneath the corresponding GSH data. The impact of co-incubation with the transition metal chelator DTPA (200 μM) is illustrated by the white bars, otherwise the figure is formatted as outlined in the legend to figure 1 with the following amendments. Notably DTPA incubation prevented not only the PM-induced loss of GSH but the background auto-oxidation seen in the particle-free controls. 'a" indicates that the concentration of GSH remaining after 4 h following co-incubation with DTPA was significantly greater than that in the 4 h particle-free control.

GSH was also depleted in a dose dependent manner by the dung cake PM extracts, 49.6 ± 4.3 and 63.5 ± 22.4% at 50 and 100 μg/m^3 ^respectively. These losses were significantly greater than those observed with ROFA, which actually demonstrated a reduced loss of GSH compared with the particle free control, or the traffic derived samples, which depleted GSH 10.2% (diesel) and 15.7% (gasoline) (Figure 2). Notably, no loss in total glutathione was noted during the incubations suggesting that adsorption to the particle surface was not occurring to any great extent. Similar to the situation with AA, co-incubation with DTPA completely abolished the loss of GSH. Incubation with DTPA also prevented the background losses of both ascorbate (-20.8 ± 6.2 μM – Figure 1) and glutathione (-47.4 ± 6.1 μM – Figure 2) from the particle-free controls over the 4 h incubation. These losses were attributable to the presence of contaminating metal ions in the synthetic RTLF following Chelex-resin treatment. Urate was not depleted from the RTLF model by any of the particle types examined in line with our previous observations [21] (data not shown).

**Figure 3:**
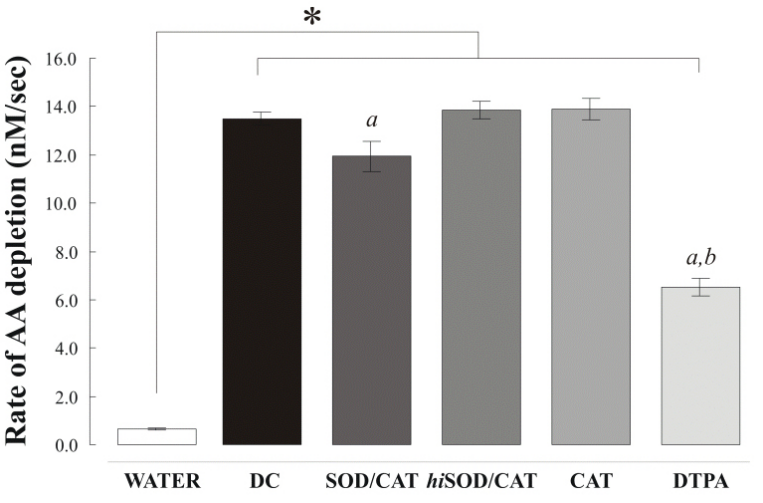
*Rate of ascorbate consumption observed from a simple ascorbate-only solution with and without a range of metal chelation and free radical scavenger treatments*. All data represent the mean (SD) of three separate experiments: AOX = particle free control; DC = dung cake samples (1–3 at 50 μg/ml); SOD/CAT = superoxide dismutase (50 U/mL) and catalase (150 U/mL); *hi*SOD/CAT = heat inactivated antioxidant enzymes; CAT = catalase only (150 U/mL), and DTPA (100 μM). 'a' indicates that the rate of ascorbate consumption was significantly reduced (P < 0.05) after SOD/CAT and DTPA treatment; 'b' that the DTPA treatment reduced the rate significantly more than the antioxidant enzyme treatment.

**Figure 4:**
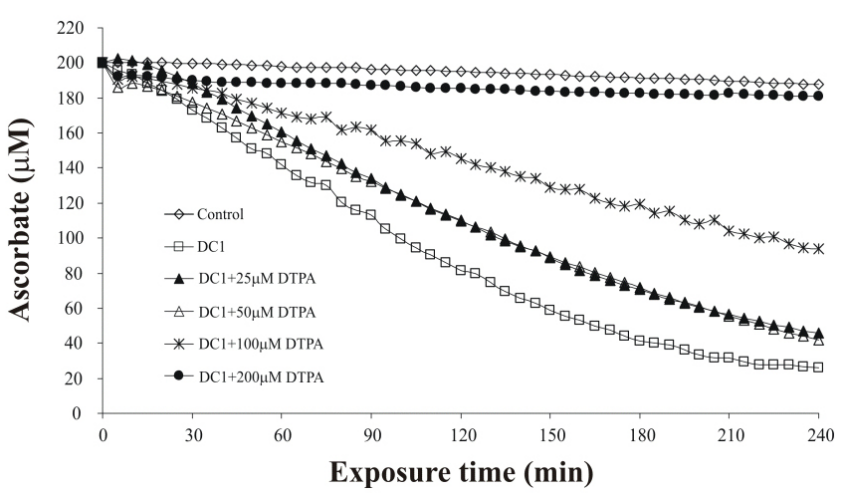
*Time dependent loss of ascorbate associated with incubation of DC at 50 μg/ml at varying concentrations of DTPA*. Each trace represents the mean of three separate experiments. The SDs on each mean value are not illustrated for graphical simplicity but were less than 5% of the mean values in all cases.

Further characterisation of the metal dependence of the oxidative reactions observed was performed using an AA only RTLF model. Individual AA consumption rates at 50 μg/mL for each of the three dung cake extracts were similar: 13.7 ± 0.15 (DC1), 13.5 ± 0.49 (DC2) and 13.3 ± 0.19 nM/s (DC3). Co-incubation with the metal chelator DTPA (100 μM) significantly reduced the rate of AA oxidation: 6.5 ± 0.36 versus 13.5 ± 0.27 nM/s for all three samples (Figure 3). Notably, in this simplified model in the absence of GSH and UA, the protection afforded by DTPA was only approximately half of that seen using the synthetic RTLF. Increasing the DTPA concentration to 200 μM completely blocked AA depletion in this model (Figure 4). Limited, though statistically significant protection was also seen using the antioxidant enzymes superoxide dismutase and catalase (11.9%), whilst no decrease in AA oxidation was seen with catalase alone or heat inactivated superoxide dismutase and catalase (Figure 3).

**Figure 5:**
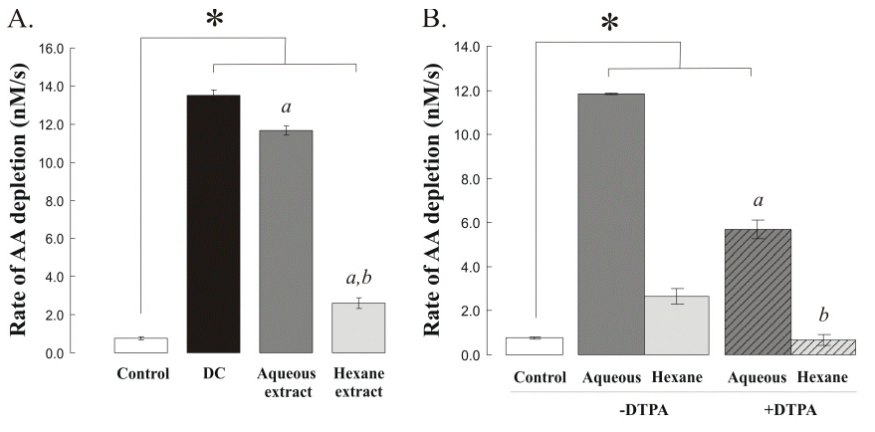
*Rates of ascorbate consumption seen with aqueous and organic extracts of dung cake (DC) particle extracts*. All data represent the mean (SD) of three separate experiments. '*' Indicates that the rate of AA depletion seen with the complete, aqueous and organic dung cake extracts was significantly greater (P < 0.05) than that observed in the particle free control; 'a' that the activity of the aqueous and organic extracts were significantly less than the complete extract; and, 'b' that the rate of AA depletion by the organic extract, with or without DTPA, was less than that seen with the aqueous fraction.

When the dung cake aqueous and organic extracts were separated the vast majority (80.6%) of the oxidative activity was found to be associated with water-soluble components (Figure 5). The residual activity associated with the hexane extract could be completely inhibited through the co-incubation of 100 μM DTPA indicating that this activity was not due to organic radicals. Consistent with these findings, dung cake aqueous extracts were shown to contain appreciable concentrations of the redox active metals Fe and Cu quantified using the chromogenic chelators bathophenantroline-disulphonate (BPS) and bathocuproine-disulphonate acid (BCS) in each of the three dung cake particle suspensions. These methods gave Fe concentrations of 7.1, 3.6, and 8.5 μg/mg in DC 1, 2 and 3 respectively and 26.2, 26.2 and 23.8 μg/mg for Cu. In comparison ROFA contained 23.7 ± 0.30 μg/mg Fe and 5.9 ± 0.73 μg/mg of Cu, whilst both metals were undetectable in the diesel and gasoline samples, using either chromogenic chelator.

## Discussion

Indoor air pollution poses a significant health risk worldwide. WHO estimates suggest that up to 6.5% of the annual disease burden in developing nations is attributable to the burning of solid fuels in the indoor environment [24,25]. Smoke from cooking stoves burning biomass fuels contains carbon monoxide, fine particulates, nitrogen dioxide and hydrocarbons; all at concentrations far in excess of what is considered unsafe in outdoor air [7]. In this study we investigated whether fine particles derived from the burning of the biofuel dung cake also displayed high levels of intrinsic oxidative activity relative to traffic and industrial derived PM. We wanted to examine the hypothesis that the health effects associated with exposure to biofuel derived PM were not solely a function of the high exposure concentrations but also because of their high content of redox active components.

It has been proposed that the capacity of inhaled particles to elicit inflammation and injury in the lung, as well as systemically, may be related to their capacity to cause oxidative stress [17]. In this working paradigm, inhaled particles generate oxidative stress through three inter-related pathways: first, by directly introducing oxidising species into the lung, such as redox active transition metals [16] or quinones [19] absorbed onto their surface. Second, by introducing surface absorbed PAHs that can undergo bio-transformation *in vivo *into quinones species through the action of the cytochrome P450, epoxide hydrolase and dihydrodial dehydrogenase detoxification pathway [26] and finally by stimulating inflammatory cells to undergo the oxidative burst. In this final case, activation of inflammatory cells may be triggered by endotoxin on the surface of inhaled particles [27], futile phagocytic processing of PM [28], or by the up-regulation of redox sensitive transcription factors directing the synthesis of pro-inflammatory cytokines [29]. The integrated sum of all these processes can be considered the 'total' oxidative activity of the particle.

In this study we measured PM oxidative activity using an *in vitro *screening procedure that assessed the capacity of PM associated pro-oxidant components (metals and quinones) to deplete physiologically relevant antioxidants, ascorbate, urate and reduced glutathione from a synthetic model of the RTLF [21,22]. This 'intrinsic' activity, measured in a cell free system, only reflects the oxidative activity attributable to redox active metals and quinone compounds and not 'latent' activities that may be associated with PAHs or endotoxin. With this caveat, we found PM samples derived from the combustion of dung cake to be significantly more active, on an equal mass basis, than either metal-rich ROFA or PAH-rich vehicle exhaust PM, despite the greater surface area of these samples. This activity was manifest by the capacity of the PM suspensions to deplete both AA and GSH from synthetic RTLF. Notably, the endotoxin and PAH content of dung cake and other biofuels have been shown to be high [24,30] suggesting that their 'total' oxidative activity is likely to be far in excess of that associated with traffic derived PM. This very high oxidative activity in animal dung combustion particles supports studies demonstrating increased pulmonary toxicity in mice following instillation of particles derived from dried municipal sewage combustion, relative to coal alone [31].

The depletion of both AA and GSH from the model was prevented by co-incubation with the metal chelator diethylenetriaminepentaacetate (DTPA) indicating that the losses observed were driven by redox active metals such as Fe, Cu, Ni, and Cr. DTPA has five acetate groups linked to a molecular backbone that permits it to form tight complexes with a broad range of metals, preventing them from catalysing damaging oxidation reactions. Desferoxamine (DFO) was not used in these studies as it has been reported to reduce chelated Cu that would have resulted in interpretive difficulties when examining its protective role in mixtures of soluble metals [32]. The contribution of superoxide and hydrogen peroxide to the observed antioxidant losses was examined in co-incubation experiments using the antioxidant enzymes Cu, Zn superoxide dismutase (SOD) and catalase (CAT). Limited protection was observed with these enzymatic antioxidants suggesting that the contribution of these reactive oxygen species to the ascorbate and glutathione losses was minor compared with those attributable to their direct oxidation during the reduction of Fe^3+ ^and Cu^2+^.

Thus we conclude that AA and GSH oxidation occurred predominately by their direct reduction of Fe^3+ ^to Fe^2+ ^and Cu^2+ ^to Cu^+^. The superoxide formed by the subsequent oxidation of ferric and cupric ions could undergo dismutation to hydrogen peroxide, reduce Fe^3+ ^and Cu^2+^, or oxidise ascorbate, urate or glutathione within the synthetic RTLF. As the reaction rate between Fe^3+ ^and superoxide (1.5 × 10^8 ^M^-1^s^-1^) greatly exceeds that its dismutation at physiological pH (5.4 × 10^5 ^M^-1^s^-1^, pH7.4) it seems likely that the former reaction predominated, especially as little loss of ascorbate or glutathione could be attributed to superoxide or hydrogen peroxide production. Interestingly, we saw no evidence of urate depletion, despite the importance of this antioxidant in protecting the airway against oxidant gases [33], peroxynitrite [34] and hydroxyl radicals [35]. The rate of the reaction of urate with hydroxyl radicals (7.2 × 10^9 ^M^-1^s^-1^, pH 6–7) is broadly similar to that of both ascorbate (1.6 × 10^9 ^– 1.1 × 10^10 ^M^-1^s^-1^, pH 7–7.4) and glutathione (9.0 × 10^9 ^– 1.3 × 10^10 ^M^-1^s^-1^, pH 8 and 7.8, respectively). Thus the absence of UA depletion in this model supported the contention that superoxide dismutation to hydrogen peroxide was not occurring to any great extent, with little evidence of hydroxyl radical generation. Whilst the redox potentials of UA and AA at pH7 (E°' = 590 and 282 mV respectively) [36] may suggest that the urate radical could be reduced back to UA at the expense of AA we do not believe that this occurred, as removal of urate from the RTLF had no impact on the observed rate of ascorbate depletion (data not shown).

We also observed that the capacity of DTPA to inhibit ascorbate oxidation was significantly reduced in the ascorbate only incubation experiment: only 100 μM being required for full inhibition in the complete synthetic RTLF as opposed to 200 μM in the ascorbate only RTLF model. This finding may imply that either GSH or UA is limiting the bioavailability of Fe, either through chelation, as has been proposed for UA [37], or by interfering with the capacity of AA to solubilise ferric iron from the particle surface [16]. We are currently investigating these potential actions of UA and GSH. Irrespective of this, when the concentration of DTPA was increased in the AA only model, all AA oxidation was blocked indicating the absence of a quinone-dependent activity in the DC particles. This contention was supported by the observation that only a fraction of the measured oxidative activity was present in organic DC extracts. Whilst other groups have emphasised the importance of PM associated quinones/hydroquinones in the oxidative activity of ambient PM [19] our data would tend to emphasise metal content as the major determinant of DC oxidative activity. Clearly the contribution of metal and organic components to PM oxidative activity may vary depending on its source. In addition it is also likely that the age and storage conditions of the filters used in this study resulted in losses of potentially reactive organic species. These cautionary caveats only further emphasise that we are probably underestimating the 'true' oxidative capacity of freshly generated DC particulates.

As these findings implicated redox active metals in the oxidation process we measured the bioavailable Fe and Cu content of the DC particles. This measurement included reduced and oxidised forms of these metals, both water-soluble and surface mobilisable through ligation to the chromogenic chelators bathophenantroline-disulphonate (BPS) [38] and bathocuproine-disulphonate acid (BCS) [39]. Using these approaches we detected considerable Fe and Cu content. The especially high content of Cu is likely to explain extensive glutathione oxidation observed with the dung cake samples, due to copper's high reactivity toward this antioxidant [18], as well as the lack of reactivity of the ROFA sample toward GSH. It should be noted, however, that the variation in the content of these metals in the three DC samples did not match their observed variation in oxidative activity implying that Fe and Cu were not the sole determinates of the observed activity. The compositional data pertaining to the ROFA sample (PM_2.5_), used in the current study have been described previously [23,40] using ICP-MS. These analyses have confirmed the relatively high concentrations of total Fe, and low concentrations of Cu in the ROFA sample derived from the burning of heavy fuel oil (N° 5). These metals are however less abundant in the ROFA sample than either vanadium (58.6 μg/g) or nickel (10.6 μg/g). In contrast, ICP-MS analysis of both the gasoline and diesel samples revealed these metals to be below detectable limits [41], which concurs with their low reactivity in this assay system and these measurements made using the chromogenic chelators. Parallel ICP-MS analysis of dung cake PM obtained under identical burn conditions has also subsequently revealed appreciable concentrations of the redox active metals Ni and Cr (45 and 40 μg/g PM, respectively) in these samples, but no detectable V. This elemental analysis will be described in detail in a subsequent manuscript.

The high metal content of the dung cake may reflect both the presence of biological metals; especially Fe and Cu in dung [42], as well as metals associated with the local soil with which the animal dung is mixed to make the bricklets. These data therefore support the initial hypothesis that fine particles derived from the controlled burning of dung cake are highly oxidative in nature due to their content of redox active metals.

## Conclusion

These data demonstrate that fine particles derived from the burning of the biomass fuel dung cake are highly oxidising. This activity appears to be largely related to their content of redox active metals, with the caveats mention above. As oxidative activity is a biologically meaningful index, the high activity associated with dung cake fine particles allied to their high concentrations in homes using dung cake fuel emphasises their potential negative health impact.

## Methods

### Sample Collection and Filter extraction

Dung-cake was obtained from Eksaal, near Mumbai. Dung cake has a high ash content, 25–30% and is made by mixing dung with mud/clay and straw to improve its mechanical strength. It is then patted down, moulded and sun-dried for 24 h, which achieves moisture content of 7–8%. As dung-cake combustion results in copious particle emissions a sampling rate of 3–4 L per minute was used in the particle sampler, and a burn time of 12 min, to ensure no clogging of the Teflon filter substrates used for particle collection. The particle sampler employed had a cyclone inlet to exclude particles larger than 2.6 μm diameter [4]. All Teflon filters were conditioned for 12-h at 50% RH and 25°C prior to weighing. Filters were shipped to the UK where the PM was extracted from the Teflon matrix using a standardized vortexing and sonication protocol into Chelex-treated water contain 5% methanol, pH 7.0 [21]. All subsequent dilutions of this particle solution were also performed in Chelex-treated water contain 5% methanol, pH 7.0. Diesel and gasoline particles in the fine mode (0.1–2.5 μm) were obtained from Professor Thomas Sandstrom (University Hospital, Umeå, Sweden) derived from idling diesel (Volvo TD45, 4.5 L, 4cylinders, 1991) and gasoline (2.0 L "Opel Omega" with a catalyst from 1989) engines. Details of the diesel fuel used have been published previously [43] whilst the gasoline engine utilized 95-octane gasoline. Residual fly ash was kindly donated by Professor Ken Donaldson (University of Edinburgh, Scotland, UK). Diesel, gasoline and ROFA PM samples were resuspended in Chelex-treated water contain 5% methanol, pH 7.0 at the required concentration and the pH of the resultant suspension adjusted to neutral pH.

### Particle Incubations and Measurement of Antioxidant Loss

For the initial experiments dung cake extracts were diluted to either 100 or 50 μg/ml and incubated at 37°C in synthetic RTLF (200 μM, AA, UA and GSH, pH7.0) for a period of 4 h. After incubation, samples were either acidified with metaphosphoric acid to a final concentration of 5% (w/v) for UA and AA measurement or diluted into 100 mM phosphate buffer for determination of GSH. Antioxidant determinations have been described previously [21]. Subsequent inhibitor studies were performed at PM concentrations of 50 μg/ml by following the loss of AA (starting concentration 200 μM, pH7.0, 37°C) at 265 nm over a 4 h time course with readings every 5 minutes. Incubations were performed with dung cake extracts only, as well as in the presence of a range of free radical scavengers and transition metal chelators: SOD/CAT = superoxide dismutase (50 U/mL) and catalase (150 U/mL); hiSOD/CAT = heat inactivated antioxidant enzymes; CAT = catalase only (150 U/mL), and DTPA = Diethylenetriamine pentaacetate (200 μM).

### Hexane extractions

1 ml of each dung cake particle suspension at 55.6 μg/ml was vortexed hard for 2 minutes with an equal volume of HPLC grade hexane. At the end of this mixing period the organic and aqueous phases were separated, and the former dried under nitrogen at room temperature. The hexane extract was then resuspended according to the standard protocol in an equal volume of Chelex-treated water containing 5% methanol.

### Total Fe and Cu determinations

Total iron concentrations in the particle suspensions were determined using the Fe^2+^-specfic chromogenic chelator, bathophenantroline disulphonate (BPS) [38]. DC particle suspensions (55.6 μg/ml) were prepared in Chelex-100 resin treated ultra-pure (18Ω) water containing 5% HPLC grade methanol (pH 7). These samples were then incubated with BPS (1 mM) in the presence of 10 mM ascorbate (final pH 4) for 30 minutes in the dark. These samples were then briefly vortexed and then centrifuged at 13,000 rpm for 15 minutes. The absorbance of the BPS-Fe complex was then determined in the particle free supernatant at 535 nm. Iron concentrations were determined against a standard curve of ferrous ammonium sulphate (0–50 μM) in ultrapure water, 5% methanol, in the presence of 10 mM ascorbic acid. Particle free and filter blanks were also ran in parallel to the extracted dung cake samples and these background concentrations subtracted from the determined dung cake Fe concentrations. A further blank containing the dung cake suspension with ascorbate but in the absence of BPS was also included and the background absorbance subtracted from the observed values in the dung cake samples. Total copper was similarly determined using the Cu+-specific chromogenic chelator bathocuproine-disulphonate at 483 nm [39], with the following amendments: the final ascorbate concentration in the samples and standards was 1 mM and the pH 7.0. Coppers concentrations were determined a standard curve of copper sulphate (0–20 μM) prepared in Chelex-100 resin treated water.

### Statistical analysis

All data are expressed throughout as means with SD. Specific experimental details are summarised the each Figure legend. Experimental group comparisons were performed using two way repeated measures ANOVA. Post-hoc comparisons of group means were performed using the Student-Newman-Kuels test. All statistical analyses were performed using SPSS for windows, version 11.5 or the Unistat Exel plug-in, version 4.53.

## List of Abbreviations used

AOX Antioxidant

AA Ascorbate

UA Urate

GSH Reduced glutathione

GSSG Glutathione disulphide

RTLF Respiratory tract lining fluid

DC Dung cake

DFO Desferoxamine

BPS Bathophenantroline-disulphonate

BCS Bathocuproine-disulphonate

DPTA Diethylene triamine pentaacetate

SOD Superoxide dismutase

*hi*SOD Heat inactivated superoxide dismutase

CAT Catalase

PAH Polyaromatic hydrocarbons

PM Particulate matter

WHO World Health Organisation

## Competing interests

**Ian S Mudway**: None

**Sean T Duggan**: None

**Chandra Venkataraman**: None

**Frank J Kelly**: None

**Jonathon Grigg**: None

## Authors' contributions

**Ian S Mudway**: Study design and experimental work, data analysis, preparation of manuscript

**Sean T Duggan**: Study design and experimental analysis

**Chandra Venkataraman**: Collection of particle samples from dung cake combustion

**Gazala Habib**: Collection of particle samples from dung cake combustion

**Frank J Kelly**: Study conception and design, manuscript review

**Jonathon Grigg**: Study conception and design
